# Unraveling the Role of Objective Food Environment in Chinese Elderly’s Diet-Related Diseases Epidemic: Considering Both Healthy Food Accessibility and Diversity

**DOI:** 10.3390/ijerph192113924

**Published:** 2022-10-26

**Authors:** Zhaohua Zhang, Yuxi Luo, Zhao Zhang, Derrick Robinson, Xin Wang

**Affiliations:** 1 School of Economics, Tianjin University of Commerce, Tianjin 300134, China; 2 School of Economics and Management, Guangxi Normal University, Guilin 541004, China; 3 College of Information and Electrical Engineering, China Agricultural University, Beijing 100083, China; 4 Aquaculture Economist, National Oceanic & Atmospheric Administration, San Diego, CA 92037, USA

**Keywords:** objective food environment, healthy aging, healthy food accessibility, healthy food diversity, lifestyle factors

## Abstract

The essential role of the objective food environment in achieving healthy aging has been widely recognized worldwide. However, the existing empirical evidence is mostly based on Western cases, and how the objective food environment associates with health outcomes among Chinese elderly remains poorly understood. By merging nationally representative micro survey data with Baidu-based spatial data on the location of food outlets, this study develops accessibility and diversity indicators to explore the relationship between food environment and diet-related diseases among Chinese elderly and investigates how healthy lifestyles moderate this relationship. The results show that improvement in healthy food accessibility and diversity decreases both the probability and the number of diet-related diseases that the elderly suffer. Having more healthy lifestyle factors is associated with a lower risk of suffering from diet-related diseases and strengthens the negative effect of healthy food environment on suffered diet-related diseases. Heterogeneity effect analysis suggests that the relationship between objective food environment and diet-related diseases differs by city scale and income level. The findings of this study shed light on designing tailor-made policies for non-Western countries to promote healthy aging.

## 1. Introduction

With the rapid aging of China’s population, the incidence of diet-related diseases (such as diabetes, hypertension, and dyslipidemia) among the elderly is increasing, which has become a major public health problem. The Annual Report on Elderly Health in China (2020–2021) showed that 58.3% of the population aged 60 and above in China suffered from hypertension, 37.2% had dyslipidemia, and 19.4% had diabetes [1]. As China’s elderly population increases, medical care for diet-related diseases has resulted in a serious economic and social burden. To address the crisis and promote construction of a healthy China, researchers and policymakers focus on exploration of factors associated with this rising prevalence of diet-related diseases.

A growing body of evidence indicates that individual-level characteristics, including genetic history [2], health behaviors [3], and dietary practice [4], do matter a great deal in determining diet-related diseases. However, the limited impact of individual-level interventions has pushed the research focus to contextual influences on health. The local food environment—defined as the geographic distribution, heterogeneity, and multiplicity of food outlets [5]—has also been recognized as a significant determinant of diet-related diseases since individual food choices and population dietary patterns could be affected by surrounding food sources [6,7]. Due to China’s rapid urbanization, the accessibility of highly processed or otherwise unhealthy foods for the elderly increases, which increases consumption of unhealthy food and augments the likelihood of suffering from diet-related diseases [8,9]. Therefore, understanding the relationship between food environment and elderly health outcomes would be helpful to improve public health and realize healthy aging.

A range of food environment measurements have been developed in the existing literature [10]. Accessibility is the most frequently studied feature of the food environment, which often involves categorizing food environments as healthy or unhealthy on the basis of food outlets [11,12]. Commonly used approaches to measure accessibility include the Retail Food Environment Index (RFEI) and the Physical Food Environment Index (PFEI), which consider the ratio of healthy food outlets to unhealthy food outlets [13,14]. However, this measure does not reflect the range and mixture of different categories of food outlets. Even with the same share of healthy food outlets, environments with a greater diversity of food outlet categories are desired. Therefore, a diversity indicator needs to be applied to convey an essential feature of the food environment that cannot be illustrated by the accessibility dimension. To reflect both the diversity of food sources and the health value of each constituent outlet category, a healthy food diversity index, which incorporates the health values of food outlets with the traditional diversity indices, is created. The health values of food outlets are developed based on the recommended dietary pattern in the Chinese Dietary Guidelines 2016 (CDG 2016) [15] and the food pyramid in this study. Furthermore, by merging nationally representative micro survey data (China Health and Retirement Longitudinal Study, CHARLS) with Baidu-based spatial data on the location of food outlets, we also explore how a healthy lifestyle moderates the relationship between food environment and diet-related diseases among the elderly. With most empirical evidence based on Western cases, the findings of this study, which focus on the Chinese context, would renew the knowledge on food environment and associated health outcomes among the elderly and provide valuable implications for designing tailor-made policies for developing countries.

The rest of this paper is organized as follows. Section 2 summarizes previous research, which is followed by a description of the data and methodology. Then, the analytical results and the main findings are presented. A discussion and conclusive remarks are presented at the end.

## 2. Previous Literature

Food environment characteristics are critical contextual factors affecting how people access food. The existing literature defines the food environment as any opportunity to obtain food, which includes physical, socio-cultural, economic, and policy influences at both micro- and macro levels [16,17]. The fundamental work of Glanz et al. [18] conceptualized the food environment consisting of the media environment, organizational environments (e.g., schools and workplaces), the community environment (i.e., type and location of stores and restaurants in neighborhoods), and the consumer environment (i.e., availability, price, and promotion of foods in stores and restaurants) [19]. Among the above dimensions of food environment, neighborhood retail food environment has been studied most extensively [20,21,22]. A large number of different measures seeking to capture the characteristics of neighborhood food environment have been employed in the existing literature. One common measure has referred to accessibility theories, which often reflect a geographical perspective of the food environment [23,24]. Approaches used to determine accessibility to different types of food outlets include the absolute number count or density [14,25], dominance of certain types of food outlets [13], path distance calculation [7], and geo-big data analysis [26]. However, among the above approaches, GIS-based accessibility and relative ratio measures are considered to be more practical given their low computational cost and reduction in same-source bias [4,27]. However, these approaches only consider the number of food outlets, overlooking the range and mixture of different categories of food outlets. A diverse diet is a cornerstone of a sufficient and balanced supply of nutrients. To test the importance of the diversity dimension of the objective food environment, we incorporate the health values of food outlets to traditional diversity indices based on the recommended dietary pattern in CDG 2016 and the food pyramid. A synthesis of both healthy food accessibility and diversity approaches should help to better evaluate the local food environment.

Food environments, which can be characterized by risk factors (i.e., exposure to high caloric foods), as well as health-promoting factors (i.e., availability of healthy food stores), can impact health. Studies examining links between the food environment and health could be classified into two categories: direct examination of the association between food environment and diet-related health outcomes (i.e., obesity, diabetes, heart disease, and high blood pressure) and indirect examination of food environment with food choice and eating behaviors. The results across studies on direct examination are mixed due to the heterogeneity in methods, measures, and debate regarding what aspects of food retail environments are most influential [28,29]. A large body of literature seeks to examine how disparities in food access and availability are related to health and demonstrates that lack of proximity to or difficulty in accessing healthy food retailers could increase the incidence of diet-related diseases [7,30]. Research on indirect examination indicated that food environment is a major determinant of food purchase and consumption choices that ultimately affect weight and diet-related diseases [2]. Hawkes et al. (2020) [31] suggested that creating healthier food environments could enable healthier dietary behaviors. Although the existing literature acknowledged the determining role of food environment on health, it is still unclear to what extent the food environment is associated with diet-related diseases among the vastly understudied but growing elderly population, and to what extent such an association is moderated through variation in individual lifestyles.

By shedding light on the association between urban food environment and health outcomes, this study extends the existing literature in three aspects: (1) measuring the objective food environment by establishing both availability and diversity indicators; (2) examining the moderating role of individual lifestyles in the relationship between food environment and diet-related health outcomes; (3) taking the understudied but growing elderly in China as the study population to provide recommendations for improving public health in a developing context.

## 3. Data and Methods

### 3.1. Data

#### 3.1.1. Diet-Related Health Outcomes

We draw on multi-source datasets for the variables used in our analysis. China Health and Retirement Longitudinal Study is a national, longitudinal survey among residents aged over 45 in China from 2008 to 2018 that includes assessment of demographic backgrounds, health status and functioning, social and economic status, and retirement information. Supported by the multistage probability sampling method, the CHARLS collected data from 150 counties/districts and 450 villages/communities from 28 provinces [32]. The latest 2018 wave survey data are applied in this study. Data about the outcome variables—diet-related diseases—are drawn from the questionnaire of health status and functioning. During the survey, participants were asked to report whether they had been diagnosed with any diet-related diseases, which include hypertension, dyslipidemia, diabetes, or high blood sugar. Our outcome variables are measured by both whether the participants had been diagnosed with any above diseases (yes = 1, no = 0) and the number of diseases they suffered from. Besides information on health status, individual demographic background and income are also included in our analysis. Information on socio-demographic characteristics of the elderly are identified from the demographic background questionnaire, which includes age, gender, and educational attainment.

#### 3.1.2. Food Environment Measures as Explanatory Variables

The food environment is evaluated from two dimensions: accessibility and diversity. Indicators used to assess these two dimensions are created based on the mixture of retail outlets as well as restaurants and fast food outlets in the city. Considering China’s retail food environment characteristics, four types of food stores are defined as healthy food outlets within the urban setting: (1) chain full-service supermarkets that supply a diversity of fresh meat, fruits, and vegetables; (2) fruit stores that offer seasonal fruits with high quality; (3) vegetation markets that provide fresh vegetables, fruits, and meat at reasonable prices; (4) seafood markets, which mainly sell fish, shrimp, crab, and other marine products. The less healthy food outlets include: (1) chain fast food shops that serve traditional Western-style hamburgers, pizza, and fried foods; (2) local fast food outlets that offer high-calorie and low-nutrient foods; (3) small and convenience stores that engage in retailing a limited line of goods, such as bread, soda, and snacks. Data for all the food outlet locations are drawn from Baidu map, which is one of the leading map service providers in China. Our data collection is based on the Baidu Places Application-Programming-Interface (API), which enables searching for place information within a specified area.

#### 3.1.3. Individual Lifestyles as Moderator

Previous studies have demonstrated that lifestyles play a vital role in explaining health and diseases [3]. A healthy lifestyle score is created to measure the healthiness of individual lifestyle in this study. Based on the lifestyle factors proposed by the American Heart Association and the Chinese cultural characteristics and lifestyles, three factors are considered, including moderate to high-intensive physical activity (≥30 min/day vs. <30 min/day); drinking alcohol (never drinking vs. drinking); smoking (never smoking vs. smoking). Diet profile is not included in this study due to data unavailability. For each healthy lifestyle factor, the participants receive 1 if they meet the criteria for health and 0 otherwise. We divide the full sample into 4 groups with the healthy lifestyle score ranged from 0 (the lowest healthy lifestyle score) to 3 (the highest healthy lifestyle score) to ensure that the sample is evenly distributed. Data on individual lifestyles are drawn from the questionnaire of lifestyle and health behaviors in CHARLS dataset.

### 3.2. Methods

#### 3.2.1. Food Environment Measures

Urban food environment is measured from two dimensions: accessibility and diversity. Regarding accessibility aspect, the modified Retail Food Environment Index (mRFEI) is applied. The mRFEI measures the percentage of healthful food retailers within the city and is calculated as follows:

(1) $$\mathrm{mRFEI}=100\times \frac{number\;of\;healthful\;food\;retailers\;in\;city}{number\;of\;healthful\;retailers\;in\;city+number\;of\;unhealthful\;retailers\;in\;city\;}$$

With regard to the diversity aspect, a modified Berry Index [33] is applied, which evaluates diversity in terms of number as well as distribution of different food items. The Berry Index (BI) is defined as:

(2) $$BI=1-\sum s_{i}^{2}$$

where $s_{i}$ is the share of item i in the total amount of food consumed. The index is bounded between 0 and $1-\frac{1}{n}$, and BI=0 if an individual consumes only 1 food item, while $BI=1-\frac{1}{n}$ if the individual consumes equal shares of all food items considered. However, from a nutritional perspective, healthy foods should be consumed in higher shares than unhealthy ones. Hence, the highest index value has to be assigned to a situation where an individual consumes food group shares recommended by food guide. Therefore, the Berry Index needs to be modified to reflect the favor of healthier foods, and the Healthy Food Diversity Index (HFDI) has been created by incorporating the health value of each type of food stores into the Berry Index. The health values are derived from actual food guideline of CDG 2016, which provides the optimal intake of each food group per day. In accordance with food pyramid, these shares are summed up in 3 groups: 69% plant foods, 29% animal foods, 2% fats and oils. Following Drescher et al. (2007) [34], each main food group (plant foods, animal foods, fats, and oils) is divided into 5 different subgroups, and the 5 subgroups are supposed to have the same heights on all sides of the food pyramid. Then, the percentage of each subgroup within the main group is obtained by geometric calculations. Combination of the subgroup shares with the main group shares yield health factors for 15 different food subgroups, which are shown in Table 1.

Based on Table 1, the health score for each type of food store is obtained based on the foods it sells. The final Healthy Food Diversity Index (HFDI) is constructed as:

(3) $$HFDI_{i}=\left(1-\sum_{j}^{N}s_{j}^{2}\right)\frac{1}{\theta }\sum_{j}^{N}hf_{j}\cdot s_{j}$$

where $HFDI_{i}$ is the Healthy Food Diversity Index for the $i_{th}$ city; $s_{j}$ is the proportion of the number of the $j_{th}$ type food outlets to the total number of food outlets within city i; $hf_{j}$ is the health score of the $j_{th}$ type food outlets; θ is a scaling parameter to ensure that $HFDI_{i}$ ranges from 0 to 1 and is calculated as the maximum value that the final summation term can take.

#### 3.2.2. Empirical Strategy

Individuals located in the same city may not be independent since they share the same objective food environment. A standard econometric method may ignore such clustering effects, resulting in downward-biased standard errors. Considering the structure of our dataset—hierarchical structure data on individuals nested within cities—the multilevel model is employed to investigate the relationship between city food environment and diet-related health outcomes among the elderly. With individuals nested within cities, the multilevel model allows the inclusion of both individual-level and city-level variables as well as different types of fixed effects. The introduction of the multilevel model controlling for the hierarchical structure of individual-level data could produce more reliable estimates. Before applying a multilevel model, the intra-class correlation coefficients (ICC), which are equivalent to a random effect ANOVA [35], are calculated to determine whether a multilevel model is required to quantify the grouping effects. The value of the ICC reflects the ratio of between-group variance to the total variance. If the average productivity and resource misallocation is independent between firms and cities, the value of ICC tends to be zero, indicating that there is no between-group difference.

The multilevel model and the ICC indicator are specified as follows:

(4) $$Y_{ij}=\gamma_{0}+\gamma_{1}X_{ij}+\gamma_{2}mRFEI_{j}+\gamma_{3}HFDI_{j}+\varepsilon_{ij}+\delta_{j}\;$$

(5) $$ICC=\frac{\;\sigma_{\delta }^{2}}{\;\sigma_{\delta }^{2}+\sigma_{\varepsilon }^{2}}$$

where $Y_{ij}$ is the diet-related health outcomes of individual i in city j, which is measured as whether the elderly have been diagnosed with any diet-related diseases and the number of diseases they suffered from; $X_{ij}$ represents the individual-level characteristics; $mRFEI_{j}$ and $HFDI_{j}$ measure healthy food accessibility and healthy food diversity of city j, respectively. $\varepsilon_{ij}$ is the individual-level residual, which is assumed to be identically independently distributed: $\varepsilon_{ij}\;\sim N\left(0,\;\sigma_{\varepsilon }^{2}\right)$. $\delta_{j}$ denotes the city-level random term satisfying $\delta_{j}\;\sim N\left(0,\;\sigma_{\delta }^{2}\right)$.

To test how individual lifestyles moderate the effect of objective food environment on diet-related health outcomes, the interactions between food environment measures and the healthy lifestyle score are introduced in Equation (4):

(6) $$Y_{ij}=\gamma_{0}+\gamma_{1}X_{ij}+\gamma_{2}mRFEI_{j}+\gamma_{3}HFDI_{j}+\gamma_{4}mRFEI_{j}*HLS_{ij}+\gamma_{5}HFDI_{j}*HLS_{ij}+\varepsilon_{ij}+\delta_{j}\;$$

where $HLS_{ij}$ is the healthy lifestyle score of individual i in city j.

## 4. Results

### 4.1. Descriptive Analysis

**Figure 1:**
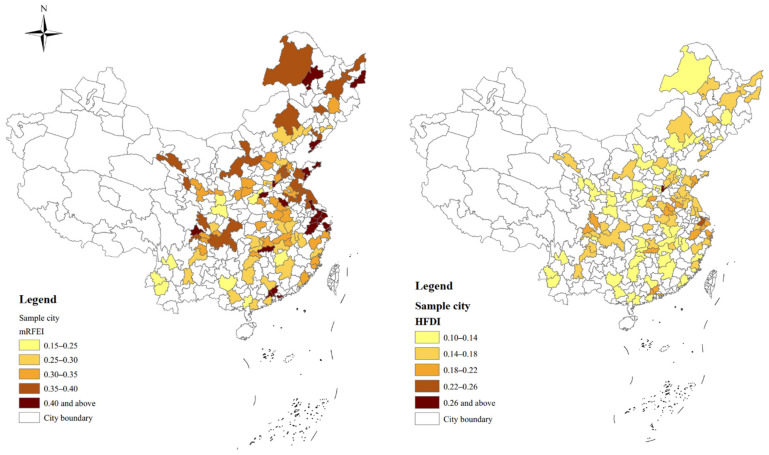
Spatial distribution of mRFEI and HFDI for sample cities.

Multi-source datasets are applied in this study. Observations from the CHARLS dataset used in this study are collected from 113 different cities. According to the sampling strategy of the CHARLS dataset, the 113 are randomly selected from 28 provinces of China, excluding Tibet Autonomous Region, Taiwan Province, Hong Kong, and Macao Special Administrative regions. Among the 113 cities, 46 of them are from the east region, 38 cities are from the central region, and 29 cities are from the west region. This aligns with city distribution among these three regions in China. The population size of these cities ranges from 1238 thousand people to 3416 thousand and covers different sizes of cities. Generally, our sample is nationally representative. With a total of 113 cities retained in this study, the spatial distributions of mRFEI and HFDI for the sample cities are shown in Figure 1. The mean value of mRFEI is 0.335, and the value for about 80% of the cities ranges from 0.25 to 0.44. The average value of HFDI is 0.155, and the value for about 80% of the cities ranges from 0.12 to 0.204. Generally, most cities with higher mRFEI and HFDI are located in developed coastal areas.

Descriptive statistics for outcome variables, individual characteristics, and city-level variables are summarized in Table 2. The outcome variables show that 44.1% of the elderly suffered at least one type of the diet-related diseases, and the mean value of the diet-related disease number is 0.7. In the sample, 51.1% of the participants are male, and the average age is 64.27. Only 6.3% of the elderly population obtained a high school degree or above. More than half of the elderly participate in physical activities more than 30 min every day, and there are fewer elderly people smoking than drinking. Besides the city food environment, which is measured both by healthy food accessibility (mRFEI) and healthy food diversity (HFDI), this study also controls city population density, gross regional product, average wage level, the number of buses, and city tier. Data for these variables were obtained from China City Statistical Yearbook, and the description and mean value for each variable are also shown in Table 2.

### 4.2. Empirical Results

#### 4.2.1. Baseline Estimates

This study aims to explore to what extent the objective food environment affects the elderly’s health status in China. Following model specification, a two-level multilevel model is applied. Prior to the empirical results, the ICC is calculated to determine whether a multilevel model is required to quantify the grouping effects. The value of the ICC reflects the contribution of between-group variance to the total variance. If the average health status is independent between individuals and cities, the value of ICC tends to be zero, indicating no need for a multilevel model. Applying the maximum likelihood method, the calculated ICCs from the null model show 20.07% of the total variation in whether suffering from diet-related diseases and 23.36% of the total variation in count number of health-related diseases are located at the city level. This result confirms the need to use a multilevel model to capture the intra-class effects. Table 3 presents the multilevel analysis results. Models 1–2 take whether the elderly population suffers from any diet-related diseases as the dependent variable, while Models 3-4 use the number of diet-related diseases the elderly suffer as the explained variable. The significantly negative coefficients of “mRFEI” in Model 1 and Model 3 indicate that improvement in the healthy food accessibility in a city reduces both the probability and the number of diet-related diseases that the elderly population suffers. On average, a 10 percent increase in healthy food accessibility reduces the risk of suffering from diet-related diseases by 25.5% and decreases the suffered diet-related disease number by 0.427. These findings corroborate the evidence in the literature that healthy food environment generates positive health outcomes [36,37]. The coefficients for “HFDI” in Model 2 and Model 4 are also significantly negative, indicating that healthy food diversity is also an important factor affecting the health status of the aging population. Cities with higher HFDI values increase the exposure of the aging population to healthy food and thereby reduce the risk of suffering from diet-related diseases. The magnitude of the coefficients in Table 3 also shows that, although both healthy food accessibility and healthy food diversity matter in determining the health status of the elderly, healthy food accessibility plays a more important role.

Most of the control variables are statistically significant and with expected signs. The individual-level control variables include gender, age, education attainment, marriage status, and income. The results in Table 3 show that older populations are associated with higher risk suffered from more diet-related diseases. Elderly with a high school degree and above have better health status than those with lower education attainment. Married elderly are less likely to suffer from diet-related diseases than single or divorced individuals, and higher income has a protective effect on the health of the elderly. However, there is an insignificant difference between male and female elderly on whether suffering from diet-related diseases or the number of diseases suffered. City-specific variables include population density, gross regional product, average wage level, the number of buses, and city tier. The results show that the health status of the elderly is better in cities with a higher population density and a higher average wage level. The gross regional product only has a significant effect on the number of suffered diet-related diseases among the elderly. City tier classified by China Business News (CBN) Weekly in 2020 is applied in this study. Besides GDP, CBN Weekly also considers other indicators to define the tier of a city, such as business resource agglomeration level, accessibility level, per capita income, population density, number of universities, and so on. All cities are classified into five tiers based on CBN Weekly. We assign a score of five to the first-tier cities and a score of one to the fifth-tier cities. Table 3 shows that the elderly living in big and developed cities have better health status. The result for the total number of public buses in the city is tricky, which shows that operating more public buses increases the incidence of diet-related diseases. The possible explanation is that the elderly in China mainly rely on public buses to travel. More buses not only improve the accessibility of healthy food but also improve the convenience of unhealthy food consumption.

#### 4.2.2. Moderating Role of Healthy Individual Lifestyles

Previous studies showed that diseases, such as hypertension and dyslipidemia, could be prevented by insisting on a healthy lifestyle [38,39]. Therefore, could the positive effect of healthy lifestyles on heath offset the negative effect of unhealthy objective food environment? To answer this question, we explored the moderating role of healthy lifestyles on the relationship between food environment and diet-related diseases. The healthy lifestyle factors mainly include smoking, physical activity, and alcohol intake in this study. Rather than evaluating these risk factors individually, this study uses the combined score of different lifestyles, which is more relevant to real-life behavior patterns, to analyze the joint impact of the overall lifestyles. The empirical results for the moderating effects of healthy individual lifestyles are shown in Table 4. Models 1–4 show that healthy lifestyles are negatively associated with the outcome variables, indicating that healthy lifestyles lower the incidence of diet-related diseases. This result confirms those of the existing literature finding that healthy lifestyles exert a direct impact on health [40,41]. The coefficients of the interaction terms between mRFEI and HLS in both Model 1 and Model 3 are significantly negative, which means that healthy lifestyles strengthen the negative effect of healthy food accessibility on both the risk of suffering from diet-related diseases and the number of diseases suffered. The coefficient of the interaction between HFDI and HLS is significant only in Model 4, demonstrating that, with the same level of healthy food diversity, the elderly with healthier lifestyles suffered from less diet-related diseases. Table 4 shows an insignificant moderating role of healthy lifestyles in the relationship between healthy food diversity and the likelihood of suffering from diet-related diseases. Generally, encouraging healthy lifestyles among the elderly would be helpful to enhance the effect of food environment improvement on health.

#### 4.2.3. Heterogeneity Effect of Food Environment by City Scale

**Figure 2:**
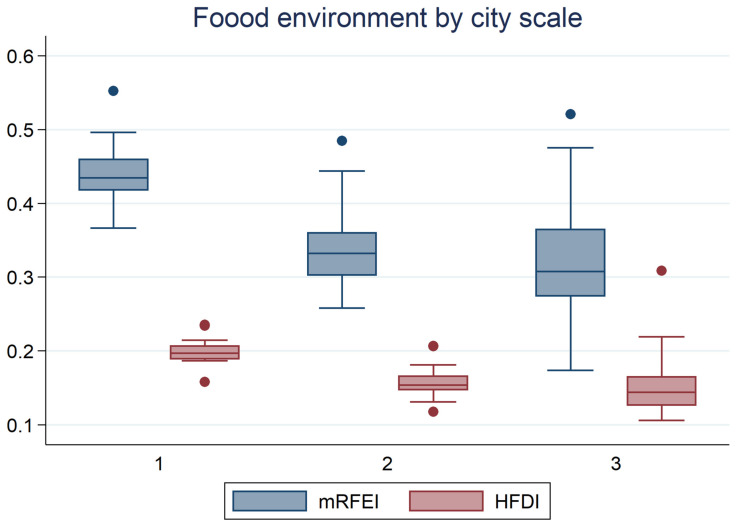
Food environment measures by city scale.

The location and type of food outlet affect the availability and accessibility of healthy food. The existing literature has shown that low-income and less developed areas are often characterized by low numbers of food outlets that sell healthy food [28,29]. Healthy dietary behaviors are more likely to occur in a supportive environment with readily accessible healthy food. Therefore, elderly living in medium and small cities are more likely to be affected by the objective environment than those living in big cities. To test this hypothesis, we divided all the cities into three categories based on the city tier: big cities, medium cities, and small cities. Big cities include the first-tier cities in the CBN Weekly classification, while medium cities include the second- and third-tier cities. Other cities are denoted as small cities. Figure 2 shows that bigger cities exhibit higher healthy food accessibility and diversity, which is aligned with previous findings. Estimated coefficients of key variables for the three subsamples are shown in Table 5. Table 5 shows that the coefficients for HLS from all three subsamples are negative and significant, indicating that, no matter if the elderly live in big cities or small cities, maintaining healthy lifestyles reduces the risk of suffering from diet-related diseases. Regarding healthy food accessibility and diversity, the result shows that, generally, elderly living in small cities benefit more from improvements in the food environment. This can be explained by the fact that, with less healthy baseline food environments in small cities, small increases in healthy food accessibility and diversity would induce high health improvement. Coefficients for interaction terms between healthy lifestyles and food environment measures show that healthy lifestyles strengthen the association of healthy food accessibility and diversity with diet-related disease outcomes in small cities. In medium and big cities, healthy lifestyles only intensify the negative effect of healthy food accessibility on suffering from diet-related diseases.

Existing studies from developed countries have shown that lower socio-economic position restricts food choices and promotes unhealthy food consumption [18]. Low income elderly in urban areas may be uniquely affected by the food environment due to financial restrictions. To test the heterogeneity effect of food environment on diet-related health outcomes among the elderly with different income levels, we further divide the elderly into three groups: low-income, medium-income, and high-income elderly based on their percentile (25%, 75%, and above) in the annual income distribution. The empirical results are shown in Table 6. First, for all three groups, healthy lifestyles are significantly and negatively associated with diet-related health outcomes. This result indicates that, regardless of income level, healthy lifestyles should be encouraged to improve health status. Comparing coefficients, it is found that healthy lifestyles play a more important role for the elderly with higher income. Improvement in healthy food accessibility significantly reduces both the likelihood and the number of diet-related diseases among the elderly with different income levels, but elderly with higher income benefit more from the improvement. With regard to the diversity dimension of the food environment, a significant effect is only found among elderly with higher income, and little evidence shows that healthy food diversity negatively impacts the diet-related health outcomes among lower income elderly. As elderly with more income are less likely to be constrained by their consumption budget, a greater diversity in healthy food outlets promotes their healthy dietary practices. Table 6 also shows that the moderating role of healthy lifestyles is significant regarding the accessibility dimension, and this moderating effect increases with income. Among medium- and high-income elderly, healthy lifestyles also strengthen the negative effect of healthy food diversity on diet-related diseases. Both the heterogeneity analysis by city scale and income level demonstrate that policies to address the needs of elderly residing in unhealthy food environments should be narrowly targeted and carefully justified.

## 5. Discussion

As the burden of diet-related non-communicable diseases among the elderly increases in both developed and developing countries, a growing body of literature has investigated the relationship between food environment and people’s health outcomes and identified food environments as one of the major drivers of the diet-related non-communicable diseases epidemic worldwide. However, studies analyzing the effects of food environments on health outcomes in developing countries are limited. Focusing on the Chinese context—a developing country with a high and further growing prevalence of diet-related diseases—this study explores the association between different aspects of food environment and diet-related diseases among the elderly. Unlike previous studies that focused on food environments of specific urban areas, we applied nationally representative data including 113 cities. Furthermore, this study produced two indicators to assess both the accessibility and diversity of healthy food in the cities to evaluate their food environments. Merging household survey data with publicly available geospatial (Baidu map) data on food retailer locations, this study provides an answer to our major research question that objective food environment does matter for suffering from diet-related diseases among the elderly. The detailed findings are as follows.

First, an improvement in healthy food accessibility and diversity decreases both the probability and the number of diet-related diseases that the elderly population suffers, which is generally in line with previous research suggesting that better access to stores that sell healthy foods was associated with better health indicators [36,37]. With the improvement in people’s living standards, more and more people realize the importance of a healthy diet. However, adherence to a diverse diet, which is recommended by the World Health Organization and the dietary guidelines for Chinese populations, has gained inadequate attention among the elderly. This explains our finding that healthy food accessibility plays a more important role than healthy food diversity in improving the elderly’s health.

Second, using a combined heathy lifestyle measure, this study shows that having more assisting healthy lifestyles is associated with a lower risk of suffering from diet-related diseases, and healthy lifestyles strengthen the negative effect of healthy food accessibility on both the likelihood of suffering from diet-related diseases and the number of suffered diseases. However, healthy lifestyles only intensify the negative effect of healthy food diversity on the number of diet-related diseases and exert an insignificant moderating role on the likelihood of suffering from diet-related diseases. Our finding is consistent with previous studies that reported a significant association between lifestyle factors and health outcomes. For example, based on survey data of 8266 individuals across China, Wang et al. (2021) [40] showed that most health-related lifestyle variables influenced individuals’ risks of overweight and obesity in urban areas; Liu et al. (2021) [42] found that suffering from chronic diseases was positively associated with unhealthy lifestyles. The significant moderating role of healthy lifestyles on the relationship between urban food environment and diet-related diseases confirms that a combination of objective food environment improvement and healthy lifestyle encouragement would be a most effective strategy to realize healthy aging.

Finally, the heterogeneity analysis suggests that the relationship between objective food environment and diet-related diseases not only differs by city scale but also varies across income levels. Specifically, elderly living in small cities benefit more from food environment improvement, and elderly with higher incomes are more sensitive to objective food environment change, especially the diversity dimension. This finding indicates that, compared to the elderly living in medium and big cities, elderly living in small cities are exposed to a food environment of low healthy food accessibility and diversity, and a small improvement in accessibility would lead to a massive improvement in health outcomes. Previous research indicated that individuals with higher income were equipped with more health-related knowledge and paid more attention to healthy diets [43]. Hence, with higher income, the elderly are willing to pay more for improvements in the food environment, especially improvement in healthy food diversity.

## 6. Conclusions

This study explores the relationship between objective food environment and health outcomes among the Chinese elderly. The empirical results provide several profound policy implications for creating an urban food environment that promotes healthy aging. First, our study suggests that policies promoting healthy aging through urban planning should focus more on healthy food provision. Although increasing the diversity of healthy food choices also reduces the consumption of unhealthy food, healthy food diversity is not recognized to be as important as accessibility among the elderly. Second, actions to improve the overall healthy food accessibility and diversity for small cities should be encouraged, such as support of community-wide vegetable and fruit supply sites and encouraging new large-chain supermarkets through tax subsidies. Additionally, it is of great concern to emphasize the importance of healthy diets and healthy lifestyles among the elderly, especially lower-income elderly. One possible option is the use of social media to promote healthy lifestyles and deliver health information to the elderly.

Due to the exploratory nature of the study, the findings of this study should be considered in light of some limitations. First, only one-year data are applied in this paper, and future research should explore how health outcomes respond to food environment dynamics using panel data. Second, classification of food outlets is based on research of developed countries. However, the dietary habits of Chinese residents are quite different from those of Western countries, and, therefore, the classification method needs to be improved in future studies. Finally, other indicators developed in this research are representative but are far from perfect, and alternatives can be further tested and compared. Despite these limitations, this study reflects the relationship between objective food environment and diet-related diseases among Chinese elderly and provides relevant suggestions for promoting healthy aging in a developing context.

## Figures and Tables

**Table 1:** Health factors for 15 food groups derived from the nutrition circle and the food pyramid of the CDG 2016.

Food Group	Share of Food Subgroup %	Health Factors
Plant foods (69%)		0.69×
Vegetables/fruits/leaf salads/juices	36	0.36 = 0.2484
Wholemeal products/paddy	28	0.28 = 0.1932
Potatoes	20	0.20 = 0.1380
White-meal products/peeled rice	12	0.12 = 0.0828
Snacks and sweets	4	0.04 = 0.0276
Animal foods (29%)		0.29×
Fish/low-fat meat/low-fat meat products	36	0.36 = 0.1044
Low-fat milk/low-fat dairy products	28	0.28 = 0.0812
Milk/dairy products	20	0.20 = 0.0580
Meat products, sausages, eggs	12	0.12 = 0.0348
Bacon	4	0.04 = 0.0116
Fats and oil (2%)		0.02×
Oilseed rape/walnut oil	36	0.36 = 0.0072
Wheat germ oil/soybean oil	28	0.28 = 0.0056
Corn oil/sunflower oil	20	0.20 = 0.0040
Margarines/butter	12	0.12 = 0.0024
Lard/vegetable fat	4	0.04 = 0.0008

**Table 2:** Descriptive statistics of the variables.

Variables	Description	Mean (Min, Max)
***Outcome variables***		
Disease dummy	Yes = 1; No = 0	0.441 (0, 1)
Disease number	Count number of health-related diseases	0.700 (0, 3)
***Personal characteristics***		
Gender	Male = 1; Female = 0	0.511 (0, 1)
Age	Age in a single year	64.27 (45, 118)
Marriage status	Legally married = 1; others = 0	0.835 (0, 1)
Education	High school and higher = 1; Other = 0	0.063 (0, 1)
Income	Annual income (¥10,000)	1.629 (0, 60)
Physical activity	Doing physical activities more than 30 min every day = 1; otherwise = 0	0.535 (0, 1)
Drinking habit	Never drinking = 1; otherwise = 0	0.549 (0, 1)
Smoking habit	Never smoking = 1; otherwise = 0	0.639 (0, 1)
***City-level Characteristics***		
mRFEI	Modified retail rood environment index (0–1)	0.335 (0, 1)
HFDI	Healthy food diversity index (0–1)	0.155 (0, 1)
Population density	Total number of population divided by city area (10,000 persons/km^2^)	5.160 (0.097, 27.59)
GRP	Gross regional product (¥100,000,000)	2.98 (0.016, 38.16)
Wage	Average wage of the city (¥10,000)	8.266 (4.525, 17.32)
Buses	Total number of buses	2512 (93, 38608)

Note: minimum and maximum values are listed in parentheses.

**Table 3:** Results of the baseline multilevel models.

Variable	Disease Dummy (Yes = 1; No = 0)		Disease Number	
	Model 1	Model 2	Model 3	Model 4
***Fixed parts***				
Gender	−0.007	−0.007	−0.010	−0.010
Age	0.004 ***	0.004 ***	0.007 ***	0.008 ***
Marriage status	−0.008 *	−0.009 *	−0.020 *	−0.021 *
Education	−0.042 **	−0.043 **	−0.063 *	−0.064 *
Income	−0.040 *	−0.036 *	−0.007 *	−0.006 *
mRFEI	−0.255 **		−0.427 **	
HFDI		−0.087 ***		−0.162 ***
Population density	−0.006 **	−0.008 ***	−0.015 ***	−0.020 ***
GRP	−0.004	−0.004	−0.013 *	−0.014 **
Wage	−0.010 *	−0.009 **	−0.017 *	−0.020 *
Buses	0.126 **	0.139 **	0.387 ***	0.408 ***
City−tier	−0.023 **	−0.022 **	−0.048 *	−0.047 **
***Random parts***				
$\sigma_{\varepsilon }^{2}$	0.241 **	0.241 **	0.999 *	0.999 *
$\sigma_{\delta }^{2}$	0.048 *	0.049 **	0.280 *	0.280 **

Note: *** *p* < 0.01; ** *p* < 0.05; * *p* < 0.1.

**Table 4:** Moderating effects of healthy individual lifestyles.

Variable	Disease Dummy (Yes = 1; No = 0)		Disease Number	
	Model 1	Model 2	Model 3	Model 4
***Fixed parts***				
Gender	0.021 *	0.021 *	0.053 **	0.052 **
Age	0.004 ***	0.004 ***	0.007 ***	0.007 ***
Marriage status	−0.009	−0.010	−0.022	−0.022
Education	0.039 **	0.039 **	0.055	0.055
Income	−0.004 *	−0.004 *	−0.008 *	−0.007 *
HLS	−0.033 *	−0.030 **	−0.024 *	−0.034 **
mRFEI	−0.243 **		−0.333 **	
HFDI		−0.087 ***		−0.134 ***
mRFEI×HLS	−0.038 *		−0.048 **	
HFDI×HLS		−0.002		−0.026 *
Population density	−0.005 **	−0.008 ***	−0.015 ***	−0.020 ***
GRP	−0.004	−0.004	−0.013 *	−0.014 **
Wage	−0.007 *	−0.009 *	−0.016 **	−0.019 *
Buses	0.128 ***	0.142 ***	0.390 ***	0.413 ***
City-tier	−0.023 **	−0.023 **	−0.049 **	−0.049 **
***Random parts***				
$\sigma_{\varepsilon }^{2}$	0.241 **	0.241 **	0.999 *	0.998 *
$\sigma_{\delta }^{2}$	0.049 *	0.044 **	0.278 *	0.258 **

Note: *** *p* < 0.01; ** *p* < 0.05; * *p* < 0.1.

**Table 5:** Heterogeneity effect of food environment by city scale.

Variable	Disease Dummy (Yes = 1; No = 0)		Disease Number	
	Model 1	Model 2	Model 3	Model 4
***Small cities***				
HLS	−0.032 *	−0.031 *	−0.042 *	−0.041 *
mRFEI	−0.248 **		−0.378 **	
HFDI		−0.108 **		−0.213 **
mRFEI×HLS	−0.066 *		−0.073 *	
HFDI×HLS		−0.016 *		−0.018 *
Controls	Yes			
***Medium cities***				
HLS	−0.023 *	−0.032 *	−0.025 *	−0.030 *
mRFEI	−0.167 *		−0.307 *	
HFDI		−0.076 *		−0.117 **
mRFEI×HLS	−0.029 *		−0.034 *	
HFDI×HLS		−0.009		0.007
Controls	Yes			
***Big cities***				
HLS	−0.003	−0.029 *	−0.018 *	−0.023 *
mRFEI	−0.080 *		−0.145 *	
HFDI		−0.049 *		−0.043 *
mRFEI×HLS	−0.021 *		−0.027 *	
HFDI×HLS		0.010		0.003
Controls	Yes			

Note: ** *p* < 0.05; * *p* < 0.1.

**Table 6:** Heterogeneity effect of food environment by income.

Variable	Disease Dummy (Yes = 1; No = 0)		Disease Number	
	Model 1	Model 2	Model 3	Model 4
***Low income***				
HLS	−0.009 *	−0.023 **	−0.014 *	−0.032 **
mRFEI	−0.052 *		−0.129 *	
HFDI		0.083		−0.061
mRFEI×HLS	−0.032 *		−0.022 *	
HFDI×HLS		−0.027		−0.016
Controls	Yes			
***Medium income***				
HLS	−0.036 *	−0.020 **	−0.034 *	−0.035 **
mRFEI	−0.172 **		−0.336 **	
HFDI		−0.039 *		−0.064 **
mRFEI×HLS	−0.026 *		−0.045 *	
HFDI×HLS		−0.049		−0.017 *
Controls	Yes			
***High income***				
HLS	−0.046 *	−0.068 **	−0.061 *	−0.044 **
mRFEI	−0.226 **		−0.349 **	
HFDI		−0.188 **		−0.248 ***
mRFEI×HLS	−0.032 *		−0.074 **	
HFDI×HLS		−0.013		−0.036 *
Controls	Yes			

Note: *** *p* < 0.01; ** *p* < 0.05; * *p* < 0.1.

## Data Availability

The CHARLS data applied in the study could be publicly obtained from the website: http://charls.pku.edu.cn/. The Baidu map data could be obtained from the website: https://lbsyun.baidu.com/apiconsole/center#/home.
